# High efficient isolation and systematic identification of human adipose-derived mesenchymal stem cells

**DOI:** 10.1186/1423-0127-18-59

**Published:** 2011-08-19

**Authors:** Xu-Fang Yang, Xu He, Jian He, Li-Hong Zhang, Xue-Jin Su, Zhi-Yong Dong, Yun-Jian Xu, Yan Li, Yu-Lin Li

**Affiliations:** 1 Key Laboratory of Pathobiology, Ministry of Education, Norman Bethune College of Medicine, Jilin University, Changchun, China; 2 Department of Pathophysiology, MuDanJiang Medical College, Hei Long Jiang, China; 3 Department of Medical Cell Biology, Uppsala University, Uppsala, Sweden; 4 Division of Orthopedics, Department for Clinical science, Intervention and technology (CLINTEC), Karolinska Institutet, Stockholm, Sweden

## Abstract

**Background:**

Developing efficient methods to isolate and identify human adipose-derived mesenchymal stem cells (hADSCs) remains to be one of the major challenges in tissue engineering.

**Methods:**

We demonstrate here a method by isolating hADSCs from abdominal subcutaneous adipose tissue harvested during caesarian section. The hADSCs were isolated from human adipose tissue by collagenase digestion and adherence to flasks.

**Results:**

The yield reached around 1 × 10^6 ^hADSCs per gram adipose tissue. The following comprehensive identification and characterization illustrated pronounced features of mesenchymal stem cells (MSCs). The fibroblast-like hADSCs exhibited typical ultrastructure details for vigorous cell activities. Karyotype mapping showed normal human chromosome. With unique immunophenotypes they were positive for CD29, CD44, CD73, CD105 and CD166, but negative for CD31, CD34, CD45 and HLA-DR. The growth curve and cell cycle analysis revealed high capability for self-renewal and proliferation. Moreover, these cells could be functionally induced into adipocytes, osteoblasts, and endothelial cells in the presence of appropriate conditioned media.

**Conclusion:**

The data presented here suggest that we have developed high efficient isolation and cultivation methods with a systematic strategy for identification and characterization of hADSCs. These techniques will be able to provide safe and stable seeding cells for research and clinical application.

## Background

Mesenchymal stem cells have been widely used in experimental and clinical research because of their unique biological characteristics and advantages [1-4]. In a previous study, we have developed standardized techniques for the isolation, culture, and differentiation of bone marrow-derived mesenchymal stem cells [5-7]. Recent reports have shown that the widely-spreaded human adipose tissue provides abundant source of mesenchymal stem cells, which can be easily and safely harvested as compared with human bone marrow [8-10]. The adipose tissue from abdominal surgery or liposuction is usually rich in stem cells which can meet the needs of cell transplantation and tissue engineering [11]. Meanwhile, these stem cells have high ability for proliferation and multilineage differentiation [12,13]. Therefore, human adipose-derived mesenchymal stem cell (hADSC) is becoming a potential source for stem cell bank and an ideal source of seeding cells for tissue engineering. Although some labs have successfully isolated hADSCs from adipose tissues, there is still no any widely-accepted efficient method for isolating and culturing highly homogenous and undifferentiated hADSCs. The comprehensive methods for identification and characterization of hADSCs have not been fully established yet. The aim of current study was to develop high efficient methods to isolate and identify hADSCs.

## Methods

### Subjects

Human adipose tissue was obtained at caesarian section from the abdominal subcutaneous tissue of obese women delivered, in the maternity department at Jilin University (age range: 23-41 years; mean = 32 years old). The subjects were healthy without any regular medication. Informed consent was obtained from the subjects before the surgical procedure. The study protocol was approved by the Ethic Committee of Jilin University. After being removed, ~5 g adipose tissue sample is relocated in a sterilized bottle filled with 0.1 M phosphate-buffered saline (PBS) at 4°C within 24 h prior to use.

### Isolation of hADSCs and Cell Culture

The procedure followed the description by Zuk et al. [14] with some modifications. The adipose tissue sample was extensively washed with sterile PBS containing 1000 U/ml penicillin and 1000 μg/ml streptomycin to remove contaminating blood cells. The specimen was then cut carefully. Connective tissue and blood vessels were removed and the tissue was cut into 1 mm^3 ^pieces. The extracellular matrix was digested with 0.1% collagenase Type I (Invitrogen, USA) at 37°C, and shaken vigorously for 60 min to separate the stromal cells from primary adipocytes. The collagenase Type I activity was then neutralized by adding an equal volume of Low glucose-Dulbecco's modified Eagle's medium (L-DMEM, Hyclone, USA) containing 10% fetal bovine serum (FBS, Invitrogen, USA). Dissociated tissue was filtered to remove debris, and centrifuged at 1500 rpm for 10 min. The suspending portion containing lipid droplets was discarded and the cell pellet was resuspended and washed twice. Contaminating erythrocytes were lysed with an osmotic buffer, and the remaining cells were plated onto 6-well plate at a density of 1 × 10^6^/ml. Plating and expansion medium consisted of L-DMEM with 10% FBS, 100 U/ml penicillin, and 100 mg/L streptomycin. Cultures were maintained at 37°C with 5% CO_2_. The medium was replaced after 48 hours, and then every 3 days. Once the adherent cells were more than 80% confluent, they were detached with 0.25% trypsin-0.02% EDTA, and re-plated at a dilution of 1:3.

### Transmission Electron Microscopy

1 × 10^7 ^hADSCs or endothelial differentiated hADSCs were washed twice in 0.1 M PBS, and then were centrifuged at 1500 rpm for 10 min. The pellet was pre-fixed in 4% glutaraldehyde at 4°C overnight, then post-fixed in 1% osmium tetroxide at 4°C for 60 min and further dehydrated in acetone and embedded in epoxy resin. Conventional ultrathin sections were prepared in Uranyl acetate. After double-stained in lead citrate, they were observed and photographed under transmission electron microscope (JEM-1200EX) (JEOL Ltd., USA).

### G-banding karyotype analysis

To analyze the karyotype of hADSCs within 12 passages, cell division was blocked in mitotic metaphase by 0.1 μg/ml colcemid for 2 h. Then the cells were trypsinized, resuspended in 0.075 M KCl solution, and incubated for 30 min at 37°C. The cells were fixed with methanol and acetic acid mixed by 3:1 ratio. G-band standard staining was used to observe the chromosome. Karyotypes were analyzed and reported according to the International System for Human Cytogenetic Nomenclature.

### Immunophenotypic Characterization

2 × 10^5 ^hADSCs were incubated with primary antibodies against human CD29, CD45, CD73, CD105, CD166, HLA-DR (Biolegend, USA) and CD31, CD34, CD44 (BD Biosciences, USA). All antibodies were diluted 1:100 and incubated with cells for 30 min at room temperature. We used same-species, same-isotype irrelevant antibody as negative control. The cells were then washed twice in PBS and incubated with fluorescein isothiocyanate (FITC)-conjugated secondary antibodies (1:50 dilution) for 30 min at 4°C. After two washing steps, cells were resuspended in 300 μl PBS for flow cytometric analysis and analyzed by fluorescein-activated cell sorting (FACS) Calibur (BD Biosciences, USA).

### Indirect Immunofluorescence assay

All hADSCs were processed as described previously [5]. Monoclonal antibodies against specific CD markers and lineage-specific proteins were used. The fluorescence signals were detected by laser scanning confocal microscope (Olympus FV500, Japan).

### Analysis of growth kinetics and cell cycle

Using cell counting, we analyzed the proliferative capacity of hADSCs from different passages. The cells were seeded onto 24-well culture plates with 5 × 10^3 ^cells per well and counted daily by trypan blue exclusion for one week and cell growth curves were recorded. The cell population doubling time (DT) of hADSCs was calculated with the Patterson formula [11]. For cell cycle anaysis, 1 × 10^7^cells were collected, fixed for 20 min at 4°C in 70% ethanol, and stained with 50 μg/ml propidium iodide (PI) at 4°C for 30 min. DNA content was analyzed by FACS Calibur using Cell Quest software (BD Biosciences, USA) in 24 h. Under these conditions, quiescent cells (G0/G1) were characterized by the minimal RNA content and uniform DNA content. The results of the study were expressed as mean ± standard error, and statistical comparisons were made using the two-sided Student's *t*-test.

### Adipogenic differentiation

Once culture-expanded cells reached ~80% confluent, they were cultured in adipogenic medium for 2 weeks. The medium consisted of L-DMEM supplemented with 10% FBS, 1 μmol/L dexamethasone, 50 μmol/L indomethacin, 0.5 mM 3-isobutyl-1-methyl-xanthine and 10 μM insulin. At the end of the culture, the cells were fixed in 4% Paraformaldehyde for 20 min and stained with Oil red-O solution to show lipid droplets in induced cells [5,13,15]. To quantify retention of Oil red O, stained adipocytes were extracted with 4% Igepal CA630 (Sigma-Aldrich, USA) in isopropanol for 15 min, and absorbance was measured by spectrophotometry at 520 nm.

### Osteogenic differentiation

The hADSCs were induced for 4 weeks in osteogenic medium containing L-DMEM, 10% FBS, 0.1 μM dexamethasone, 200 μM ascorbic acid, 10 mM β-glycerol phosphate [5]. After induction, osteoblasts were confirmed by cytochemical staining with alkaline phosphatase (ALP) to detect the alkaline phosphatase activity, and then were stained with 40 mM Alizarin Red S dye (pH 4.2) to detect mineralized matrix according to the protocol described previously [16,17]. Phosphatase Substrate Kit (Pierce, IL, USA) containing PNPP (*p*-nitrophenyl phosphate disodium salt) was used to quantify the ALP activity in cell cultures. PNPP solution was prepared by dissolving two PNPP tablets in 8 ml of distilled water and 2 ml of diethanolamine substrate buffer. Cells were plated at 5000 per well in 96 well plates and cultured in OBM for 2 weeks. After washing twice with PBS, cells were incubated with 100 μl/well PNPP solution at room temperature for 30 min. 50 μl of 2 N NaOH was added to each well to stop the reaction. Non-cell plated wells treated by the same procedure were used as blank control. The absorbance was measured at 405 nm in a kinetics ELISA reader (Spectra MAX 250, Molecular Devices, CA, USA).

### Semi-quantitative RT-PCR

Osteogenic or adipogenic specific marker-osteopontin (OPN) or PPARγ-2 gene expression was detected by semi-quantitative reverse transcriptase-polymerase chain reaction (sqRT-PCR). Total RNA was extracted from uninduced hADSCs and induced hADSCs with Trizol reagent (Invitrogen, USA). Using total RNA as template, reverse transcription reactions were carried out with oligo dT-adaptor primer. Then semi-quantitative PCR amplification was performed for human OPN and PPARγ-2. The primers used are listed below: OPN specific primers, 5'-CCAAGTAAGTCCAACGAAAG-3' and 5'-GGTGATGTCCTCGTCTGTA-3'; PPARγ-2 specific primers, 5'-CATTCTGGCCCACCAACTT-3' and 5'-CCTTGCATCCTTCACAAGCA-3'; β-actin specific primers, 5'-CATGTACGTTGCTATCCAGGC-3' and 5'-CTCCTTAATGTCACGCACGAT-3'. PCR cycles were as follows: 94°C for 2 minutes, (94°C for 30 seconds, 55°C for 30 seconds, 72°C for 1 minute) × 35 cycles, 72°C for 5 minutes. The PCR products were analyzed by electrophoresis on 1.5% agarose gel and image acquisition and data analysis were accomplished with Digital Gel Image System (Tanon, China).

### Endothelial differentiation and immunocytochemical analysis

Endothelial differentiation was induced as described previously with some modifications [18-20]. A 24-well cell culture plate was coated with fibronectin (FN) (5 μg/cm^2^) (BD Bioscience, USA) in each well. 1 × 10^4 ^hADSCs were seeded in plates and incubated for up to 15 days in endothelial differentiation medium containing endothelial growth medium (EGM2-MV) (Lonza, USA) supplemented with 50 ng/mL vascular endothelial growth factor-165 (VEGF_165_) (PeproTech, USA), 100 U/mL penicillin, and 100 μg/mL streptomycin. 15 days after endothelial differentiation started, the cells were fixed with 4% paraformaldehyde for 10 min at room temperature, and rinsed with PBS. The fixed cells were then incubated for 1 hour at 37°C with mouse antibodies against human CD31 or CD34 (BD Bioscience, USA), KDR (NeoMarker, USA) at 1:500 dilution. After incubation in a blocking solution containing 1% normal goat serum, they were incubated with secondary antibodies. A streptavidin-biotin peroxidase detection system was used to detect antibody binding.

## Results

### Isolation method gave high yield of hADSCs with normal morphological characters

**Figure 1:**
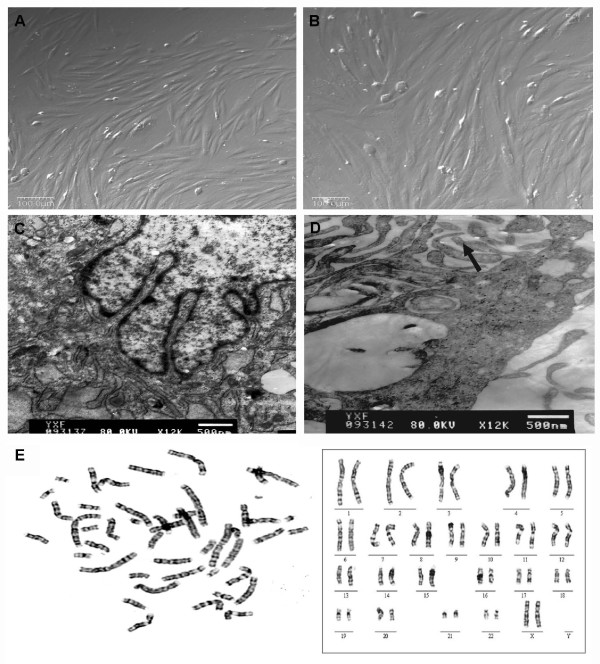
**The morphological features and karyotype of hADSCs**. The hADSCs are typical fibroblast-like cells with fusiform shape from the 3rd passage (P3) (A) to the 12th passage (P12) (B) (Bars = 100 μm). Under transmission electron microscope, hADSCs exhibited irregular nuclear morphology and abundant organelles (C), abundant microvilli with some inclusion body-like structures (arrow) (Bars = 500 nm) (D). (E) One of two reports from G-banding karyotype analysis at P12 showed normal female chromosome type: 46, XX.

The hADSCs were isolated from human adipose tissue by collagenase digestion. One gram of adipose tissue could give yield up to 1 × 10^6 ^hADSCs. They were passaged every 4-5 days for a maximum of 12 passages without major morphological alteration. The primary and passaged cells all displayed typical fibroblast-like morphological features with fusiform shape (Figure 1A, B).

Under the transmission electron microscope, most of the hADSCs showed irregular morphology of nuclear located at one side of the cell, and the cytoplasm contained numerous mitochondria and rough endoplasmic reticulums (Figure 1C). Abundant microvilli extended from cell surface into the cytoplasm and formed inclusion body-like structures (Figure 1D).

Karyotypes of two hADSC cultures were analyzed and reported according to the International System for Human Cytogenetic Nomenclature. Both results showed normal female chromosome type (46, XX) with no chromosome abnormalities observed (Figure 1E).

### The cells from different passages expressed same MSC-specific markers

**Figure 2:**
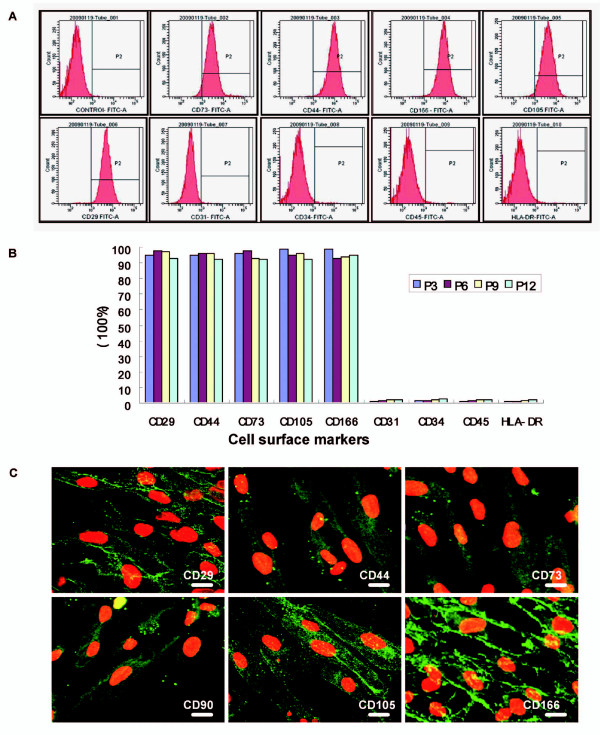
**The hADSCs expressed a unique set of CD markers**. (A) Flow cytometry analysis disclosed that the 3rd passage (P3) were positive for CD29, CD44, CD73, CD105 and CD166 with expression rates all up to 95%, but negative for CD31, CD34, CD45 and HLA-DR. (B) This immunophenotype was consistent among different passages. (C) Merged images from immunofluorescent staining of CD antigens (green) and propidium iodide (PI) staining of nuclei (red) demonstrated the same phenotype (Bars = 10 μm).

To characterize the hADSCs population, CD marker profile was examined. About 95% cells expressed CD29, CD44, CD73, CD105 and CD166, which are accepted as markers for mesenchymal stem cells [14] (Figure 2). In contrast, the hematopoietic lineage markers CD31, CD34, and CD45 were not detected. Additionally, the major histocompatibility complex (MHC) class II (HLA-DR) antigen was also negative (Figure 2A). There was no statistical difference in the expression of these markers among all 12 passages (Figure 2B).

After indirect immunofluorescent staining, hADSCs were observed by laser confocal scanning microscope. Cells that were assayed with monoclonal antibodies against the 6 MSC-specific markers showed green fluorescence, which confirmed the results above (Figure 2C).

### Growth kinetics indicated high capacity of proliferation

**Figure 3:**
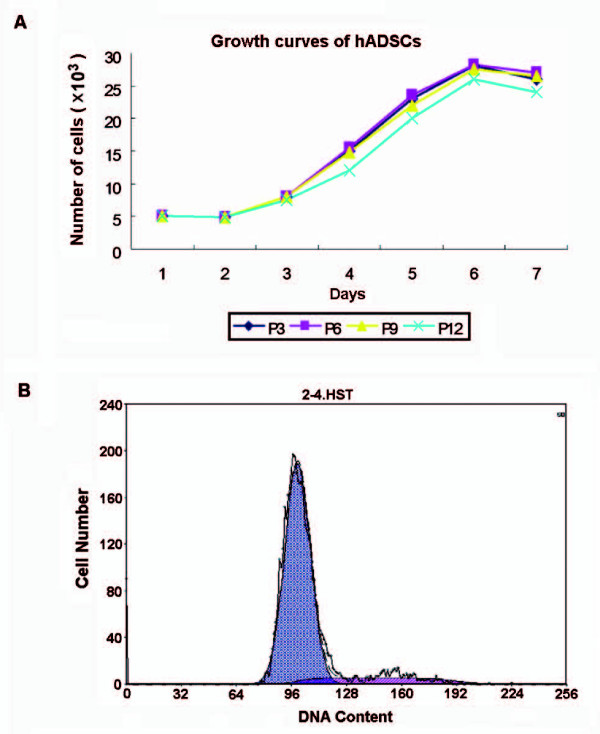
**Growth kinetics and cell cycle analysis**. (A) The growth curves showed no significant difference in the growth rate among different passages. (B) 15.1 ± 2.9% of the cells was in S+G2/M phase (active proliferative phase) (pink area) with the remaining cells in G0/G1 phase (quiescent phase, 84.9% ± 2.9%) (blue area).

The growth kinetics of viable hADSCs was determined by cell counting with trypan blue exclusion method. All of the growth curves from different passages displayed an initial lag phase of 2 days, a log phase at exponential rate from 3 to 5 days, and a plateau phase. According to the Patterson formula, the doubling time in the log phase of the 3rd passage was 24.8 hours. There was no significant difference in the growth rate among different passages (Figure 3A). The DNA content was analyzed by FACS Calibur and the cell cycle was analyzed with the Cell Quest software. The result showed that 15.1 ± 2.9% of the cells was in S+G2/M phase (active proliferative phase) with the remaining cells in G0/G1 phase (quiescent phase, 84.9% ± 2.9%) (Figure 3B).

### The hADSCs had good mutilineage differentiate potential

**Figure 4:**
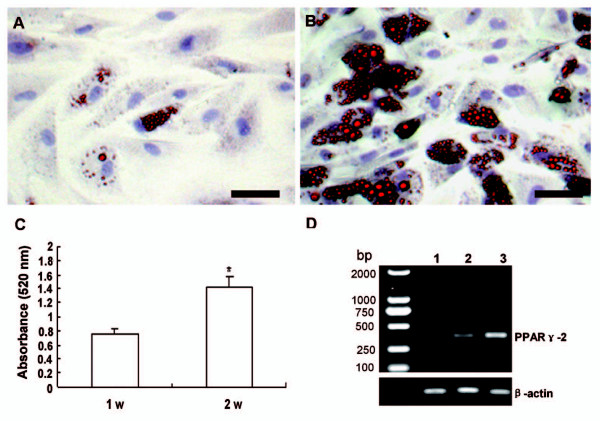
**Adipogenic differentiation of hADSCs**. The lipid was detected by Oil-red O staining after induced for 1 week (A) and 2 weeks (B) (Bars = 100 μm). (C) Quantification of the adipogenesis was done by extraction of the Oil red O retention. *P < 0.01. (D) The expression of adipogenic specific marker PPAR-γ was detected by sqRT-PCR, lane 1: non-induced hADSCs control; lane 2: hADSCs induced for 1 week; lane 3: hADSCs induced for 2 weeks.

After adipogenic induction for 3 days, the cell morphology changed from long spindle-shape into a round or polygonal shape. One week later, small bubble-shaped oil red O-staining lipid droplets appeared in part of the cells (Figure 4A). The size of lipid droplets increased after two weeks, and most of the differentiated cells showed red lipid droplet throughout the cytoplasm (Figure 4B). After induction for 2 weeks, adipocyte number increased in time-dependent manner, which is confirmed by Oil red O staining followed by retention quantification (Figure 4C). hADSCs being induced for 2 weeks displayed higher expression of the PPAR-γ mRNA than cells that had been induced for 1 week, which confirmed the oil red O staining results (Figure 4D).

**Figure 5:**
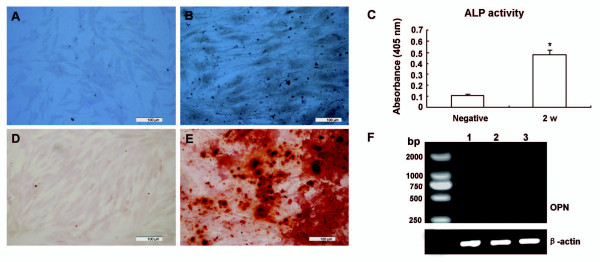
**Osteogenic differentiation of hADSCs**. Compared to non-induced control (A), Alkaline phosphatase staining was increased after being induced for 2 weeks (B) (Bars = 100 μm) and ALP activity was quantified by PNPP analysis (C). *P < 0.01. Calcium nodule formation was demonstrated by Alizarin red staining (D: non-induced control; E: induced for 4 weeks) (Bars = 100 μm). (F) The expression of the osteogenic specific marker osteopontin (OPN) was detected by sqRT-PCR, lane 1: non-induced hADSCs control; lane 2: hADSCs induced for 2 weeks; lane 3: hADSCs induced for 4 weeks.

When hADSCs were cultured in osteogenic medium for 2 weeks, osteoblast-like cells could be clearly demonstrated by alkaline phosphatase (ALP) staining (Figure 5A, B) and ALP activity was increased as shown by PNPP quantification (Figure 5C). *In vitro *mineralization could be shown at later stage (4 weeks) by Alizarin red staining (Figure 5D, E). A time-dependent increase of another osteoblastic marker, osteopontin, was shown with semi-quantitative RT-PCR analysis (Figure 5F).

**Figure 6:**
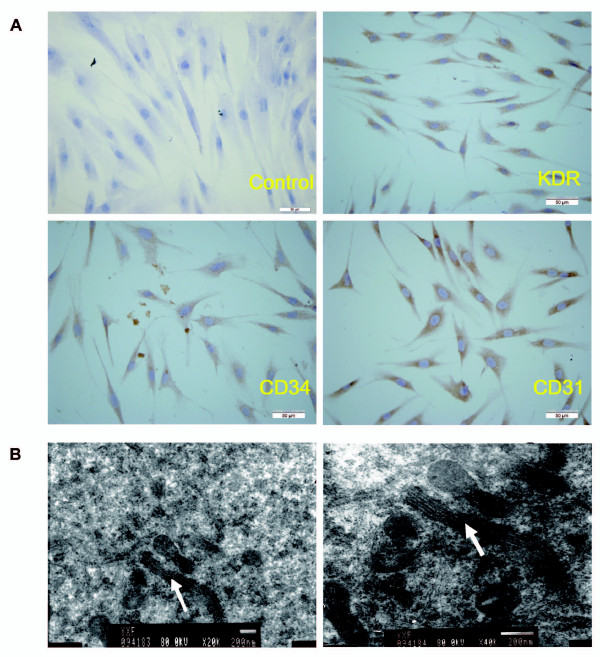
**Immunocytochemical analysis and ultrastructure of hADSCs under endothelial differentiation**. (A) The expression of endothelial-specific protein vascular endothelial growth factor receptor-2 (KDR), CD34 and CD31 were detected by diaminobenzidine staining of the secondary antibody (Bars = 50 μm). (B) Ultrastructural images showed clear specific endothelial granule, the Weibel-Palade body (arrow) (Bars = 200 nm).

After hADSCs had been cultured in endothelial differentiation medium for 15 days, these cells were evaluated for markers of endothelial differentiation. Immunocytochemical analysis confirmed their endothelial phenotype with expression of known endothelial cell markers including CD31, CD34, and KDR. In contrast, undifferentiated cells did not express any of them (Figure 6A). Additionally, Weibel-Palade body, the specific endothelial granule, was also observed by transmission electron microscopy (Figure 6B).

## Discussion

Seeding cell is one of the key elements in tissue engineering. Recent reports have shown that hADSCs can be easily harvested from adipose tissue without ethical concern or problems of transplant rejection, and these cells have high proliferation rates for *in vitro *expansion with multilineage differentiation capacity [8-13]. Because of these favorable characteristics, there is considerable interest in the applications of hADSCs. Since Rodbell first isolated preadipocytes from adipose tissue [21] a variety of methods have been developed, but the purity of isolated hADSCs is not high and the methods for identification have not been fully developed. Therefore, developing high efficient methods to isolate and identify hADSCs would be very valuable.

As demonstrated in the present manuscript we have established a simple and effective way to obtain high-purity hADSCs by using collagenase digestion and adherence screening. Isolated hADSCs proliferated at a high rate and maintained a multipotentdifferentiation capacity *in vitro *for up to 12 passages.

Since no unique molecular marker for mesenchymal stem cells has been established we used multiple surface markers for hADSCs identification. Mesenchymal stem cells bind to extracellular matrix through surface antigens which involve in cell-cell and cell-matrix interactions [22], we therefore selected adhesion molecules, including CD44, CD166, CD29 (a member of the integrin family), and mesenchymal markers (such as CD73 and CD105). The results showed that the positive staining rate was 95% or more, and the hematopoietic/leukocytic/endothelial markers such as CD31, CD34, CD45 and the major histocompatibility complex (MHC) class II (HLA-DR) were negative. These data not only excluded endothelial cell contamination, but also suggested that the clinical application of hADSCs can bypass MHC restriction. Consequently they were suitable for allograft procedures, consistent with the report of Aust [23]. In addition, the phenotypes of hADSCs showed no significant difference between different passages, indicating that the cells can be stably amplified *in vitro *for several passages. Ultrastructural imaging suggested that hADSCs were quite active with high capacity of protein synthesis and nutrients uptake as reported before [24]. Most cells were in resting period of cell cycle agreeing with the characteristics of human bone marrow-derived mesenchymal stem cells [5]. The doubling time was also consistent with stem cell characteristic, namely, a high degree of proliferation. No chromosomal abnormalities were observed in hADSCs of passage 12, providing an experimental basis for the safely clinical application of these cells. Furthermore, our studies showed that hADSCs could differentiate into osteoblasts, adipocytes and endothelia, which are typical mesenchymal stem cell characteristics.

## Conclusions

Taken together, this study developed an efficient method for isolation and cultivation of a large amount of hADSCs. It also established a systemic and comprehensive strategy to identify and characterize these cells. These data will significantly contribute to tissue engineering by providing abundant seeding cells with high quality.

## List of abbreviations

ADSCs: adipose-derived mesenchymal stem cells; MSCs: mesenchymal stem cells; ALP: alkaline phosphatase; DT: doubling time; EGM2-MV: endothelial cell growth medium 2; FACS: fluorescein-activated cell sorting; FBS: fetal bovine serum; FITC: fluorescein isothiocyanate; FN: fibronectin; KDR: kinase insert domain receptor; L-DMEM: low glucose-Dulbecco's modified Eagle's medium; MHC: major histocompatibility complex; OPN: osteopontin; PBS: phosphate-buffered saline; PI: propidium iodide; sqRT-PCR: semi-quantitive reverse transcriptase-polymerase chain reaction; VEGF_165_: vascular endothelial growth factor-165.

## Competing interests

The authors declare that they have no competing interests.

## Authors' contributions

XFY and XH carried out the cell culture and drafted the manuscript. JH conducted the complementary experiments. LHZ did immunofluorescence and immunocytochemical assays. XJS was in charge of flow cytometric analysis. ZYD took part in differentiation assays. YJX by part initiated the study. YL and XH participated in manuscript modification. XH and YLL conceived the study, organized the experimental schedule and conducted the manuscript writing. All authors have read and approved the final version of the manuscript.

## References

[B1] Barry FP Murphy JM Mesenchymal stem cells: clinical applications and biological characterization Int J Biochem Cell Biol 2004 36 568 584 10.1016/j.biocel.2003.11.001 15010324

[B2] Jiang Y Jahagirdar BN Reinhardt RL Schwartz RE Keene CD Ortiz-Gonzalez XR Reyes M Lenvik T Lund T Blackstad M Du J Aldrich S Lisberg A Low WC Largaespada DA Verfaillie CM Pluripotency of mesenchymal stem cells derived from adult marrow Nature 2002 418 41 49 10.1038/nature00870 12077603

[B3] Pittenger MF Mackay AM Beck SC Jaiswal RK Douglas R Mosca JD Moorman MA Simonetti DW Craig S Marshak DR Multilineage potential of adult human mesenchymal stem cells Science 1999 284 143 147 10.1126/science.284.5411.143 10102814

[B4] Pittenger MF Mosca JD McIntosh KR Human mesenchymal stem cells: progenitor cells for cartilage, bone, fat and stroma Curr Top Microbiol Immunol 2000 251 3 11 10.1007/978-3-642-57276-0_1 11036752

[B5] He X Li YL Wang XR Guo X Niu Y Mesenchymal stem cells transduced by PLEGFP-N1 retroviral vector maintain their biological features and differentiation Chin Med J (Engl) 2005 118 1728 1734 16313759

[B6] Lennon DP Caplan AI Isolation of human marrow-derived mesenchymal stem cells Exp Hematol 2006 34 1604 1605 10.1016/j.exphem.2006.07.014 17046583

[B7] Ng AM Kojima K Kodoma S Ruszymah BH Aminuddin BS Vacanti AC Isolation techniques of murine bone marrow progenitor cells and their adipogenic, neurogenic and osteogenic differentiation capacity Med J Malaysia 2008 63 121 122 19025015

[B8] Izadpanah R Trygg C Patel B Kriedt C Dufour J Gimble JM Bunnell BA Biologic properties of mesenchymal stem cells derived from bone marrow and adipose tissue J Cell Biochem 2006 99 1285 1297 10.1002/jcb.20904 16795045 PMC4048742

[B9] Kern S Eichler H Stoeve J Kluter H Bieback K Comparative analysis of mesenchymal stem cells from bone marrow, umbilical cord blood, or adipose tissue Stem Cells 2006 24 1294 1301 10.1634/stemcells.2005-0342 16410387

[B10] Yoshimura H Muneta T Nimura A Yokoyama A Koga H Sekiya I Comparison of rat mesenchymal stem cells derived from bone marrow, synovium, periosteum, adipose tissue, and muscle Cell Tissue Res 2007 327 449 462 10.1007/s00441-006-0308-z 17053900

[B11] Jurgens WJ Oedayrajsingh-Varma MJ Helder MN Zandiehdoulabi B Schouten TE Kuik DJ Ritt MJ van Milligen FJ Effect of tissue-harvesting site on yield of stem cells derived from adipose tissue: implications for cell-based therapies Cell Tissue Res 2008 332 415 426 10.1007/s00441-007-0555-7 18379826 PMC2386754

[B12] Bunnell BA Estes BT Guilak F Gimble JM Differentiation of adipose stem cells Methods Mol Biol 2008 456 155 171 10.1007/978-1-59745-245-8_12 18516560

[B13] Strem BM Hicok KC Zhu M Wulur I Alfonso Z Schreiber RE Fraser JK Hedrick MH Multipotential differentiation of adipose tissue-derived stem cells Keio J Med 2005 54 132 141 10.2302/kjm.54.132 16237275

[B14] Zuk PA Zhu M Ashjian P De Ugarte DA Huang JI Mizuno H Alfonso ZC Fraser JK Benhaim P Hedrick MH Human adipose tissue is a source of multipotent stem cells Mol Biol Cell 2002 13 4279 4295 10.1091/mbc.E02-02-0105 12475952 PMC138633

[B15] Zuk PA Zhu M Mizuno H Huang J Futrell JW Katz AJ Benhaim P Lorenz HP Hedrick MH Multilineage cells from human adipose tissue: implications for cell-based therapies Tissue Eng 2001 7 211 228 10.1089/107632701300062859 11304456

[B16] Halvorsen YD Franklin D Bond AL Hitt DC Auchter C Boskey AL Paschalis EP Wilkison WO Gimble JM Extracellular matrix mineralization and osteoblast gene expression by human adipose tissue-derived stromal cells Tissue Eng 2001 7 729 741 10.1089/107632701753337681 11749730

[B17] Liu G Zhou H Li Y Li G Cui L Liu W Cao Y Evaluation of the viability and osteogenic differentiation of cryopreserved human adipose-derived stem cells Cryobiology 2008 57 18 24 10.1016/j.cryobiol.2008.04.002 18495102

[B18] Chen MY Lie PC Li ZL Wei X Endothelial differentiation of Wharton's jelly-derived mesenchymal stem cells in comparison with bone marrow-derived mesenchymal stem cells Exp Hematol 2009 37 629 640 10.1016/j.exphem.2009.02.003 19375653

[B19] Ferreira LS Gerecht S Shieh HF Watson N Rupnick MA Dallabrida SM Vunjak-Novakovic G Langer R Vascular progenitor cells isolated from human embryonic stem cells give rise to endothelial and smooth muscle like cells and form vascular networks in vivo Circ Res 2007 101 286 294 10.1161/CIRCRESAHA.107.150201 17569886

[B20] Tao J Sun Y Wang QG Liu CW Induced endothelial cells enhance osteogenesis and vascularization of mesenchymal stem cells Cells Tissues Organs 2009 190 185 193 10.1159/000218139 19420896

[B21] Rodbell M The metabolism of isolated fat cells. IV. Regulation of release of protein by lipolytic hormones and insulin J Biol Chem 1966 241 3909 3917 4288359

[B22] Lange C Schroeder J Stute N Lioznov MV Zander AR High-potential human mesenchymal stem cells Stem Cells Dev 2005 14 70 80 10.1089/scd.2005.14.70 15725746

[B23] Aust L Devlin B Foster SJ Halvorsen YD Hicok K du Laney T Sen A Willingmyre GD Gimble JM Yield of human adipose-derived adult stem cells from liposuction aspirates Cytotherapy 2004 6 7 14 10.1080/14653240310004539 14985162

[B24] Akino K Mineta T Fukui M Fujii T Akita S Bone morphogenetic protein-2 regulates proliferation of human mesenchymal stem cells Wound Repair Regen 2003 11 354 360 10.1046/j.1524-475X.2003.11507.x 12950639

